# Genomic Insights Into the Body Size Evolution in Mustelidae (Mammalia: Carnivora)

**DOI:** 10.1002/ece3.72286

**Published:** 2025-10-20

**Authors:** Tian Xia, Guolei Sun, Guangshuai Liu, Chao Zhao, Lei Zhang, Xibao Wang, Xiufeng Yang, Xiaoyang Wu, Xiaodong Gao, Honghai Zhang

**Affiliations:** ^1^ College of Life Sciences Qufu Normal University Qufu China

## Abstract

The extraordinary body size diversity within Mustelidae makes this carnivoran family an exceptional model for investigating adaptive evolution, yet the genomic underpinnings of their morphological variation have remained largely unexplored. Herein, we conducted a comprehensive comparative genomic analysis of 19 mustelid species to investigate the genetic foundations underlying the extraordinary range of body sizes within this family. We accounted for phylogenetic relatedness in our analyses to avoid statistical non‐independence due to shared ancestry and identified 149 genes significantly associated with body mass variation across the mustelid phylogeny (body size‐associated genes, BAGs) and, through selection pressure analyses, further detected 125 positively selected and 409 rapidly evolving genes in large‐bodied mustelid lineages. We found that body size evolution in mustelids is driven by convergent positive selection in metabolic, developmental, and cytoskeletal pathways, with genes involved in growth signaling and energy metabolism underlying the remarkable morphological diversity observed across the family. Our study reveals the genetic basis of body size evolution in mustelids, offering important insights into the core mechanisms driving adaptive phenotypic diversity among mammals.

## Graphical abstract

The comparative genomic analysis of 19 mustelid species provides insights into the genetic basis of body size evolution, identifying candidate genes and pathways under selection that underlie the remarkable morphological diversity in Mustelidae.

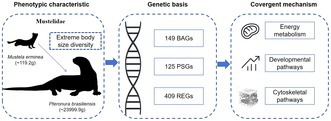

## 1 Introduction

Mustelidae is the most species‐rich family in the order Carnivora, comprising 59 recognized species classified into 22 genera across the subfamilies Mustelinae, Melinae, Lutrinae, and Taxidiinae (Yonezawa et al. 2007). Members of the Mustelidae family display a remarkable ecological diversity and a wide array of locomotor adaptations, ranging from fossorial species like badgers to semi‐ or fully aquatic ones such as otters (Koepfli and Wayne 2003). These species typically exhibit streamlined bodies, short limbs, and shortened snouts, as seen in animals such as weasels (*Mustela* spp.) and minks (*Neogale* spp.). Wolverines ( *Gulo gulo* ) and badgers ( *Meles meles* ), on the other hand, display more robust body structures (Hutchings and White 2000). Previous theories posit that the evolution of elongated body forms may have facilitated the exploitation of new resources during the Mid‐Miocene epoch, thereby contributing to the enhanced species diversity observed among “elongate” mustelids (Law et al. 2018). The slender, elongated body structure of mustelids likely enhanced their capacity to infiltrate burrows and maneuver through confined spaces to capture prey, and is believed to have contributed to the clade's proliferation and subsequent diversification (Sato et al. 2012).

Body size represents a central trait in macroecology and macroevolutionary studies, serving as a key functional proxy for understanding organism‐environment interactions (Smith et al. 2016). Body size governs fundamental organismal traits that shape the architecture and functioning of ecological networks across hierarchical scales (Woodward et al. 2005). Body size evolution, as a pivotal adaptive trait, has been systematically investigated across diverse vertebrate lineages through phylogenetic comparative frameworks, such as Primates (Harcourt and Schreier 2009), Carnivora (Huang et al. 2021), and Cetacea (Sun et al. 2019), among others. Recent research on mustelid body size has attracted significant scientific attention, as exemplified by a recent study examining the relative influences of ecological and phylogenetic factors. While classical eco‐morphological frameworks have emphasized diet and climate, accumulating macroevolutionary evidence shows that aquatic or semi‐aquatic habitats are associated with larger body size in mammals (Gearty et al. 2018; Farina et al. 2023). Interestingly, unlike classical eco‐morphological theories that link body size with diet or climate, this mustelid‐focused study using phylogenetic comparative methods found that semi‐aquatic habitat specialization independently promotes body size enlargement, thereby challenging traditional assumptions about macroevolutionary drivers in mammalian lineages (Rodrigues et al. 2023).

Mustelidae, the most diverse family within Carnivora, exhibits remarkable interspecific variation in body size, ranging from small weasels to large otters, reflecting their wide ecological diversity and morphological specializations (Rodrigues et al. 2023). This exceptional diversity in both species number and size makes mustelids particularly well‐suited for investigating the evolutionary determinants of body size. Despite increasing attention to the ecological drivers of mustelid body size evolution, the regulatory mechanisms underlying mustelid‐specific size diversification remain critically underexplored at molecular levels. While the ongoing improvements in the quality and availability of mustelid genomes offer new opportunities to explore the mechanisms underlying the remarkable body size diversity in this group, in this study, we conducted comparative genomic analyses using 19 high‐quality mustelid genomes to provide new insights into the molecular mechanisms driving body size evolution in this family.

## 2 Methods

### 2.1 Phenotype and Genome Data Collect

Body mass is a principal morphological trait widely used to assess size variation, behavioral adaptations, and evolutionary trajectories across animal species (Herberstein et al. 2022). Nineteen mustelid species, with body masses ranging from 119.4 to 23,999.9 g, were selected to represent the body size diversity within Mustelidae. The body mass data were collected from the Phylacine 1.2 platform (Faurby et al. 2018). We then divided these mustelid species into three groups‐ large (body mass > 23,500 g), small (body mass < 140 g), and medium‐sized (remaining species) for subsequent analysis. High‐quality genomes of 19 mustelids and one out‐group dog ( *Canis lupus familiaris* , GenBank accession number: GCF_011100685.1) were downloaded from the NCBI database (Table 1).

**TABLE 1:** Genomic and body mass information of 19 mustelid species.

Species	Accession numbers	Genome size	Scaffold N50	Genome coverage	Assembly level	Mass (g)
*Neovison vison*	GCF_020171115.1	2.7 Gb	220.3 Mb	21×	Chromosome	945
*Mustela erminea*	GCF_009829155.1	2.4 Gb	130.1 Mb	62.86×	Chromosome	119.4
*Mustela nivalis*	GCA_964662115.1	3.4 Gb	115.4 Mb	33×	Chromosome	137.5
*Mustela lutreola*	GCF_030435805.1	2.6 Gb	154.1 Mb	36.15×	Chromosome	440
*Mustela nigripes*	GCF_022355385.1	2.5 Gb	145.4 Mb	85×	Chromosome	850
*Mustela eversmanii*	GCA_963422785.1	2.5 Gb	64.3 Mb	56×	Scaffold	1350
*Mustela putorius*	GCF_011764305.1	2.6 Gb	23.6 Mb	183.7×	Contig	915.4
*Pteronura brasiliensis*	GCA_004024605.1	2.6 Gb	119 kb	47.7×	Scaffold	23999.9
*Lontra canadensis*	GCF_010015895.1	2.4 Gb	18.5 Mb	40×	Scaffold	8087.4
*Lutra lutra*	GCF_902655055.1	2.4 Gb	149 Mb	63×	Chromosome	8785.1
*Enhydra lutris*	GCF_002288905.1	2.5 Gb	38.8 Mb	110×	Scaffold	23,500
*Eira barbara*	GCA_020311275.1	2.5 Gb	42.5 Mb	55.34×	Scaffold	3910
*Gulo gulo*	GCA_024510155.1	2.4 Gb	144 Mb	96.87×	Scaffold	17012.6
*Martes zibellina*	GCA_012583365.1	2.4 Gb	5.2 Mb	114×	Scaffold	1130
*Martes martes*	GCA_963455335.1	2.5 Gb	146.3 Mb	35×	Chromosome	1300
*Martes flavigula*	GCA_029410595.1	2.4 Gb	143.1 Mb	107.96×	Chromosome	1842.5
*Meles meles*	GCF_922984935.1	2.7 Gb	133 Mb	85×	Chromosome	13,000
*Mellivora capensis*	GCA_004024625.1	3.1 Gb	59.1 kb	60×	Scaffold	8500
*Taxidea taxus*	GCA_003697995.1	2.4 Gb	54.6 kb	44×	Scaffold	7107.6

### 2.2 Phylogenetic Tree Construction and Divergence Time Estimation

To infer the phylogenetic relationships of Mustelidae lineages, we generated the datasets from the genomes and performed a phylogenetic analysis to clarify their evolutionary history. Firstly, whole‐genome alignments were created from 19 mustelid genomes and one out‐group relative species with LAST (version 956) (Kiełbasa et al. 2011) and MULTIZ (version 10.6) (Blanchette et al. 2004). Then, high‐confidence “one‐to‐one” orthologous genes were detected using OrthoFinder (version 2.4.0) with the diamond algorithm among all involved genomes of mustelids. The nucleotide sequences of orthologues were aligned with MUSCLE (version 3.8.91), and alignments of coding sequences were generated with pal2nal (version 14.1) and then combined to build an independent data matrix used for phylogenetic tree construction. The maximum likelihood (ML) tree was constructed using RAxML (8.2.12) with the parameters: ‐m GTRGAMMA ‐f a ‐x 12,345 ‐N 100 ‐p 12,345 ‐o dog. The Bayesian relaxed‐molecular clock method, performed in the MCMCtree program, was used to estimate the divergence time within Mustelidae. Two calibration timepoints (dog vs. Mustelidae: ~48.5–43.4 Mya; genus *Neovison* vs. genus *Mustela*: ~11–6 Mya) were used as constraints in the MCMCtree estimation.

### 2.3 Identification of Body Size Associated Genes (BAGs)

To account for phylogenetic relatedness when examining trait‐gene associations, we used phylogenetic generalized least squares (PGLS), implemented in the “Caper” package in R, to test the potential associations between the evolutionary rates of each gene and each phenotypic trait (body mass) (Orme et al. 2013). For the PGLS analyses, an ultrametric phylogenetic tree of 19 mustelid species was obtained from the above analyses. Root‐to‐tip *ω* values offer a more holistic representation of a gene's evolutionary trajectory, rendering them particularly suitable for regression analyses with phenotypic traits in extant mustelid species. All root‐to‐tip *ω* values and body mass data underwent log10 transformation to improve data normality for regression analyses. The analyses were performed under a Brownian motion model, and the phylogenetic signal (*λ*) was estimated using the maximum likelihood method, with *λ* serving as a quantitative measure of the phylogenetic signal (Pagel 1999). A *λ* value close to 1 indicates a strong phylogenetic signal in the data.

### 2.4 Selective Pressure Analysis

The orthologues database was used to estimate positive selective evolution and rapid evolution using codeml in PAML (version 4.10.7). The evolutionary rate ratio (*ω*, Ka/Ks) of terminal branches was chosen as an indicator to estimate the selection pressures. First, we performed branch model (one‐ratio model and two‐ratio model) to test for rapidly evolving genes (REGs) at the foreground branch. The null model constrained the *ω* to remain constant across all phylogenetic branches, while the alternative model allowed for branch‐specific variation in *ω* values, specifically along the foreground lineages. The likelihood ratio test (LRT) was employed to statistically compare the models, with a Bonferroni‐corrected *p*‐value cutoff of 0.05 used to determine significant REGs. Then, branch‐site model (Model A vs. Model A null) was used to test for positively selected genes (PSGs). The null hypothesis constrained the *ω* ratio to 1 for every site across all branches, while the alternative hypothesis permitted site‐specific *ω* variation exclusively in the foreground lineage. We set the target branch (large and small) as the foreground branch, respectively, and the remaining branch as the background branch. PSGs were identified using a nominal *p*‐value threshold of 0.05 and required at least one positively selected site (posterior probability > 0.90) based on empirical Bayesian analysis.

## 3 Results

### 3.1 Phylogenetic Tree Construction and Divergence Time Estimation

**FIGURE 1:**
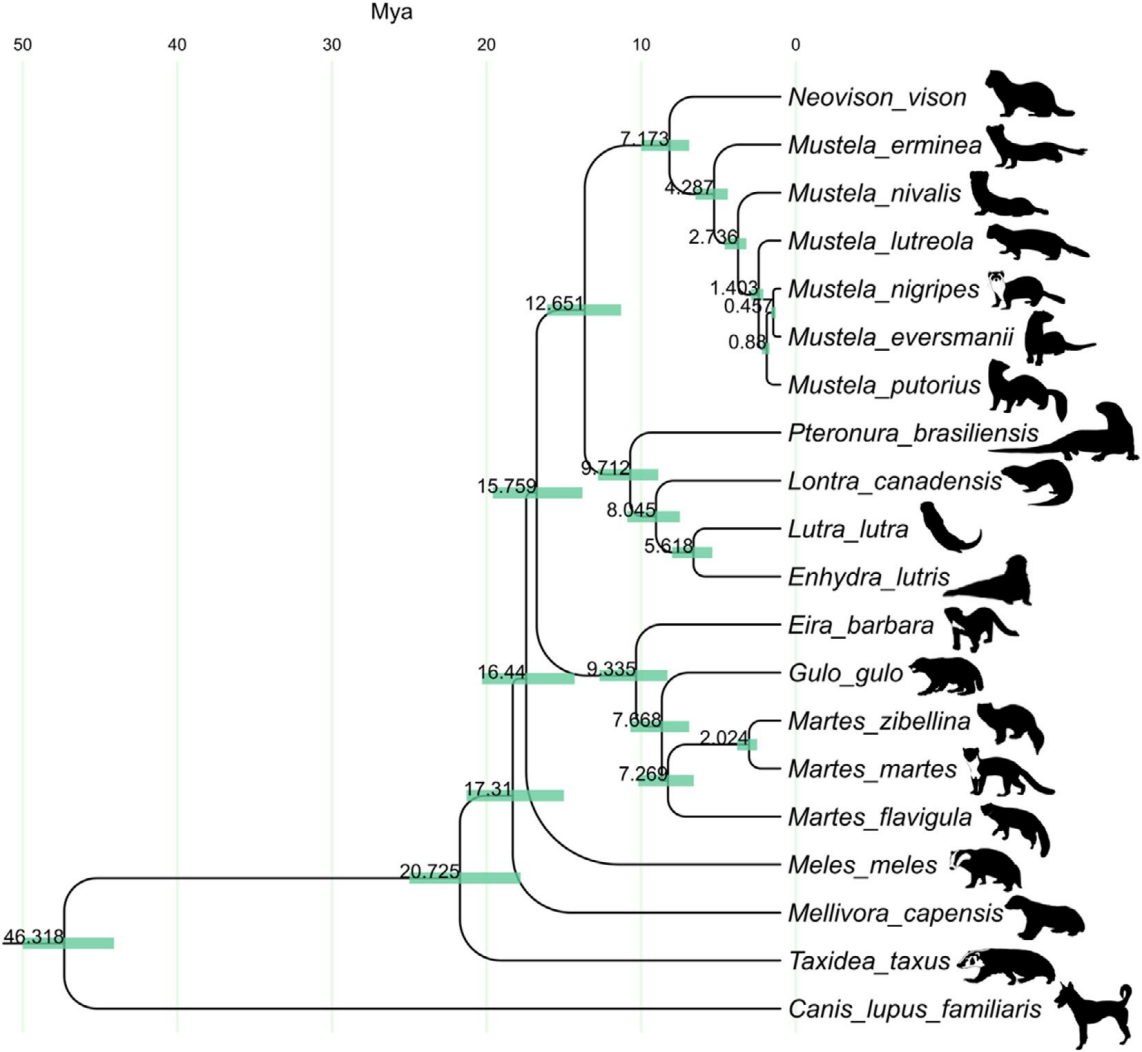
Phylogenetic tree and divergence times of mustelid species.

A total of 15,122 one‐to‐one orthologous genes were identified in the 19 mustelids and one dog (canid) genome by Orthofinder. The mustelid evolutionary tree reveals basal splits in honey badgers (*Mellivora*) and American badgers (*Taxidea*), followed by European badgers (*Meles*), a mid‐diverging tayra‐wolverine‐marten clade, and later radiations of otters (Lutrinae) and *Mustela* weasels, with *Mustela* showing recent rapid diversification. Our phylogenetic results indicate that Mustelidae originated around 25.6 million years ago (Mya) (95% CI: 24.0–16.8 Mya). The earliest divergences within the family involved the genera *Taxidea*, *Mellivora*, and *Meles* between 20.7 and 16.4 Mya. The lineage leading to otters (Lutrinae) separated from other mustelids at approximately 13.1 Mya (95% CI: 15.1–10.3 Mya). *Eira* and the clade containing *Gulo*, *Martes*, and *Mustela* diverged at 9.3 Mya, and the genus *Mustela* split from its relatives around 7.2 Mya (95% CI: 9.0–5.9 Mya) (Figure 1).

### 3.2 Genome Scanning of BAGS

**FIGURE 2:**
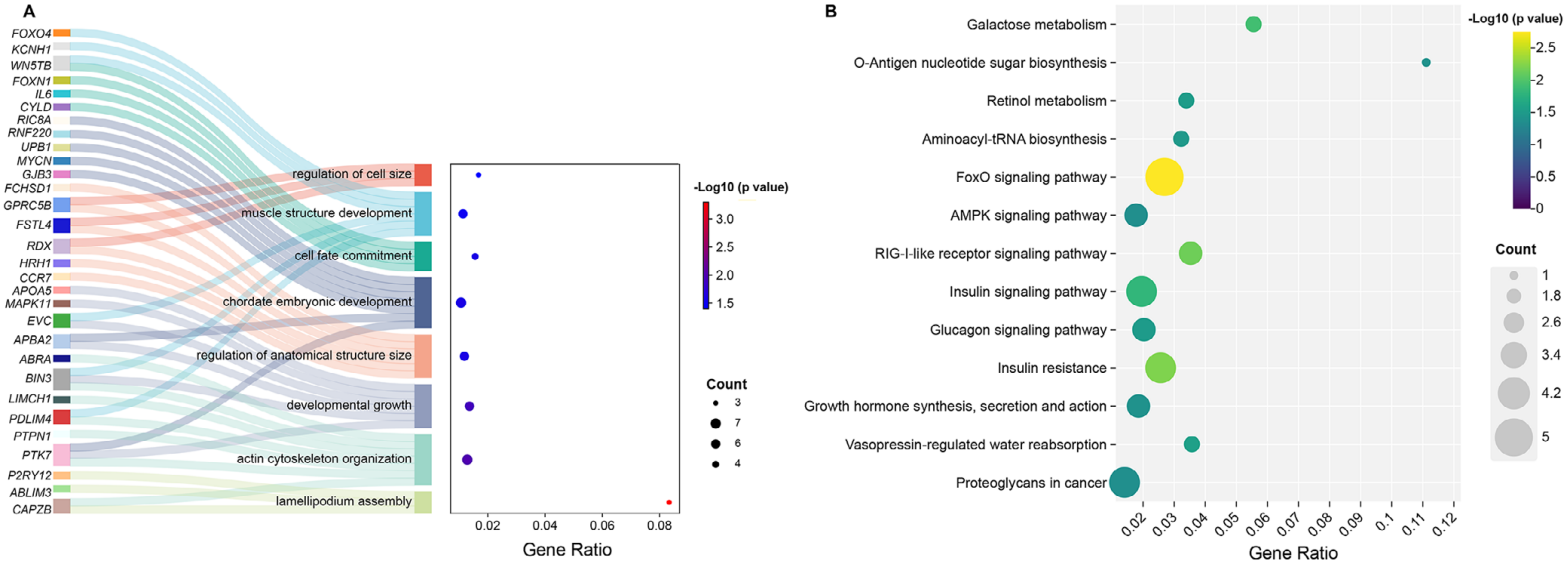
Enrichment plots of BAGs: (A) Sankey plot of GO enrichment for BAGs; (B) KEGG enrichment plot for BAGs.

PGLS analyses identified 149 genes significantly correlated with body mass among mustelids (Table S1). Functional enrichment analyses indicated that these significantly related BAGs were enriched (*p* < 0.05) in 227 GO terms, comprising 173 biological processes, 25 cellular components, and 29 molecular functions. These genes were especially enriched in processes related to growth and development, such as developmental growth (GO:0048589), cell fate commitment (GO:0045165), chordate embryonic development (GO:0043009), and muscle structure development (GO:0061061), and regulation of cell and anatomical structure size (GO:0008361 and GO:0090066). Several terms pertained to cytoskeleton organization and cell projection assembly (e.g., actin cytoskeleton organization (GO:0030036), lamellipodium assembly (GO:0030032)), which are fundamental for cell shape, migration, and tissue morphogenesis (Figure 2A).

BAGs were also enriched in metabolic processes, including amino acid (GO:0006520), lipid (GO:0016042), fatty acid (GO:0006631), and carbohydrate metabolism (GO:0016052), which highlights their role in supplying energy and biosynthetic materials during growth. Key signaling pathways involved in growth regulation (MAPK (GO:0043409) and Wnt (GO:0030177)) were significantly enriched, as were pathways related to hormonal regulation (insulin secretion (GO:0050796), steroid hormone response (GO:0048545)) and multicellular organismal‐level homeostasis (GO:0048871). Enrichment was also observed for cellular component and molecular function categories, particularly those involved in cell migration (actin cytoskeleton (GO:0015629), cell–cell junction (GO:0005911)) and metabolic activity (actin binding (GO:0003779), transporter (GO:0015103), and catalytic activities (GO:0043085)) (Table S2).

The KEGG pathway enrichment analysis of BAGs revealed significant enrichment in multiple biological pathways (Figure 2B). Our results showed notable enrichment in carbohydrate metabolism pathways including galactose metabolism (ko00052) and O‐antigen nucleotide sugar biosynthesis (ko00541). BAGs were also significantly associated with retinol metabolism (ko00830), a crucial pathway for growth and development, and aminoacyl‐tRNA biosynthesis (ko00970), essential for protein translation and cellular growth. Several signaling cascades were prominently enriched, particularly those regulating energy homeostasis and growth, including the FoxO signaling pathway (ko04068), AMPK signaling pathway (ko04152), and RIG‐I‐like receptor signaling pathway (ko04622). Notably, multiple hormone‐related pathways were identified, including insulin signaling pathway (ko04910), glucagon signaling pathway (ko04922), insulin resistance (ko04931), and growth hormone synthesis, secretion, and action (ko04935). The enrichment of vasopressin‐regulated water reabsorption (ko04962) suggests involvement of BAGs in fluid homeostasis. Additionally, we observed significant enrichment in proteoglycans in cancer (ko05205), indicating potential roles of these genes in cell proliferation and tissue organization. These findings collectively highlight the diverse molecular mechanisms underlying body size variation, spanning metabolic processes, signaling pathways, and hormonal regulation.

### 3.3 PSGs in Different Body Sizes Groups

**FIGURE 3:**
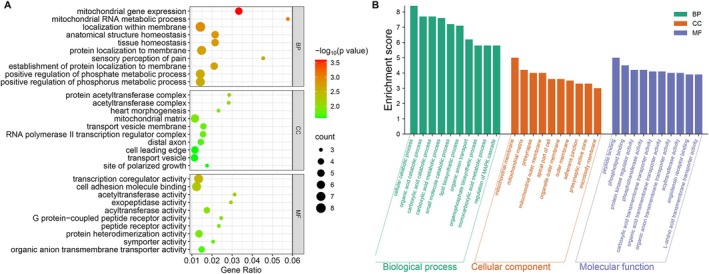
GO enrichment plots of PSGs and REGs: (A) GO enrichment plot for PSGs; (B) GO enrichment plot for REGs.

We identified 125 orthologous genes under positive selection in the two large‐bodied ( *Pteronura brasiliensis* and *Enhydra lutris* ) mustelid species (Table S1). Functional enrichment analyses revealed that these PSGs are significantly involved in pathways crucial for energy metabolism (such as mitochondrial gene expression (GO:0140053), fatty acid metabolic process (GO:0006631), oxidoreductase activity (GO:0016491)), and tissue development (including heart morphogenesis (GO:0003007), gland development (GO:0048732), and regeneration (GO:0031099)) essential for supporting increased body size. Enrichment in mitochondrial translation (GO:0032543) and rRNA processing (GO:0006364) likely reflects the elevated energy demands of larger tissues, while regulation of the actin cytoskeleton (GO:0032956) and membrane protein localization (GO:0072657) ensures structural stability during growth. Key GO terms included tissue (GO:0001894) and anatomical structure homeostasis (GO:0060249), heart development (GO:0007507), actin cytoskeleton organization (GO:0032956), cell adhesion (GO:0050839), and membrane organization (GO:0061024), reflecting genetic adaptations for organ integrity, vascular function, and efficient tissue structure in large‐bodied mustelids. Pathways related to secretion (GO:0046903) and transmembrane transport (GO:0008514, GO:0005342 etc.) indicate adaptations in nutrient distribution and intercellular communication (Figure 3A and Table S3).

In contrast to large‐bodied mustelids, which emphasize energy metabolism and tissue architecture, small‐bodied PSGs are more involved in amino acid metabolism, including the modified amino acid biosynthetic process (GO:0042398), amino acid biosynthetic process (GO:0008652), and non‐proteinogenic amino acid metabolic process (GO:0170041), as well as cellular organizational processes, particularly those related to cytoskeletal regulation (GO:0032956, regulation of actin cytoskeleton organization; GO:1902903, regulation of supramolecular fiber organization; GO:0051495, positive regulation of cytoskeleton organization). Transport processes also feature prominently, including axonal transport (GO:0098930), axo‐dendritic transport (GO:0008088), and cytoskeleton‐dependent intracellular transport (GO:0030705), underlining the importance of precise intracellular trafficking and neural function. Furthermore, enriched signaling and response pathways, such as cellular response to growth factor stimulus (GO:0071363), response to fibroblast growth factor (GO:0071774), and response to steroid hormone (GO:0048545), suggest a strong role for fine‐tuned growth and stress adaptation, with additional selective pressures on membrane organization and targeting (GO:0045121, membrane raft; GO:0016324, apical plasma membrane; GO:0090313, regulation of protein targeting to membrane). Enrichment for terms related to neuronal structure and function, such as axon cytoplasm (GO:1904115), dendritic spine (GO:0043197), and regulation of synaptic transmission (GO:0050806), along with developmental processes like tube formation (GO:0035148) and embryonic development (GO:0009792), indicate adaptations that may support neural specialization, agility, and rapid developmental transitions in small‐bodied species (Table S4).

### 3.4 REGs in Large Body Size Mustelids

Using the branch model in PAML, we identified 409 rapidly evolving genes in lineages with extremely large body sizes (Tables S1 and S3), of which ten overlapped with BAGs (Table 2, *TOMM40L, AQP5, PRKAG3, HMGB4, POLDIP3, ABCD4, IQCB1, RDX, LOC131820621*, and *LOC131820471*). The GO enrichment results for rapidly evolving genes are shown in Figure 3B. Through sequence alignment on the UniProt online platform (https://www.uniprot.org/blast), we annotated the LOC131820621 and LOC131820471 genes as olfactory receptor (OR) genes. This conclusion is based on their high sequence similarity to known olfactory receptors in other species and the presence of conserved structural domains characteristic of OR family proteins. Among these ten genes, the *RDX* gene has undergone strong positive selection in large‐bodied mustelids.

**TABLE 2:** List of overlapping genes between BAGs and REGs.

Gene	*ω* values of background	*ω* values of foregroud	Lnl (one‐ratio model)	Lnl (two‐ratio model)	*p*
*ABCD4*	0.082	0.24694	−3353.217564	−3350.80851	0.028162242
*AQP5*	0.0414	0.15238	−1719.410337	−1717.4668	0.048659129
*HMGB4*	0.1367	1.86349	−1386.719867	−1382.799128	0.005106084
*IQCB1*	0.23781	0.73426	−3834.322229	−3831.878312	0.027046598
*POLDIP3*	0.09576	0.27573	−3311.169742	−3308.604049	0.023496958
*PRKAG3*	0.1797	0.57185	−4329.532388	−4325.61753	0.005139417
*RDX*	0.01019	0.10268	−3295.84972	−3292.528831	0.009961447
*TOMM40L*	0.04276	0.22692	−1730.646275	−1728.164408	0.025884257

## 4 Discussion

Understanding the morphological trajectories of body size evolution is essential for unraveling the genetic and developmental mechanisms that generate adaptive phenotypic diversity across species (Ozgul et al. 2010). As the most diverse family in Carnivora, Mustelidae exhibits remarkable morphological adaptations such as elongated body plans, miniaturization, and limb reduction, which are key drivers of their ecological and evolutionary success (Law 2019). While molecular mechanisms underlying body size variation have been extensively studied in other vertebrates, the genetic basis of extreme body size diversification among mustelids remains poorly explored. In this study, we integrated comparative genomics with integrative bioinformatics strategies to systematically investigate the molecular mechanisms underlying body size evolution in mustelids.

The phylogenetic results are largely consistent with previous findings based on mitochondrial and nuclear genes regarding the phylogenetic relationships of mustelids (Law et al. 2018), validating their evolutionary history at the whole‐genome level. Our phylogenetic analysis of mustelid species, with divergence time estimates spanning from the Miocene to the Quaternary, provides important insights into the evolutionary history of this carnivoran family. These findings complement and extend previous molecular phylogenetic studies of mustelids (Koepfli et al. 2008) and support the hypothesis that major paleoenvironmental and climatic shifts during the Neogene and Quaternary periods significantly influenced mustelid diversification patterns. The first major burst of generic diversification, resulting in the emergence of *Taxidea* (approximately 20.7 Mya), *Mellivora* (approximately 17.3 Mya), as well as the clade that includes *Meles, Gulo, Martes, Eira*, Lutrinae, and Mustelinae, occurred firmly in the Late Miocene (around 12–8 Mya). This timing closely follows the Mid‐Miocene Climatic Optimum (17–15 Mya) and coincides with sustained global cooling, and Antarctic and early Arctic ice‐sheet build‐up (Zachos et al. 2001). By the early Late Miocene, fossil evidence shows that Eurasia had developed a more heterogeneous mosaic of vegetation types compared to earlier periods (Behrensmeyer 1992), and these shifts in plant communities likely promoted mustelid diversification through mechanisms such as geographic isolation, divergent habitat selection, and the formation or reorganization of ecological niches. A second, Pliocene pulse of genus‐level radiations (e.g., Lutrinae at ~9.7 Mya, *Mustela* at ~5.5 Mya and *Martes* at ~2.0 Mya) matches the onset of high‐latitude glaciation, expanded steppe taiga biomes and explosive diversification of small prey such as muroid rodents and passerines (Koepfli et al. 2008).

However, the evolution of mustelid body size represents a complex process shaped by multiple factors, including genetic components. Body mass is a multifaceted continuous trait regulated by diverse genes, gene families, and pathways. Notably, different genes may experience distinct selective pressures, as evidenced by the varied evolutionary signatures across functional categories in our analysis. The genomic mechanisms underlying body size evolution in Mustelidae reveal complex interactions between metabolic, developmental, and structural adaptations. BAGs were significantly enriched in growth and development pathways, including developmental growth, cell fate commitment, and the regulation of cell, tissue, and anatomical structure size, suggesting their potential roles in regulating body size variation. The enrichment of developmental processes such as cell fate commitment and tissue morphogenesis aligns with findings in cetaceans and carnivorans (Sun et al. 2019; Huang et al. 2021). Genes associated with the developmental growth pathway have been shown to be differentially expressed among different sheep breeds, highlighting the important role of this pathway in Dorper sheep development and meat quality (Peng et al. 2022). Our finding that key signaling cascades (MAPK and Wnt) are identified among BAGs aligns with their established roles as master regulators of body and organ size (Gokhale and Shingleton 2015; Arnold et al. 2019). Mitogen‐activated protein kinases (MAPK) and members of the Wnt family function as key signaling molecules in the regulation of cell proliferation, homeostasis, and development, and both are crucial for morphogenesis, tissue patterning, and skeletal development (Zhang et al. 2014). Our analyses also reveal the integral role of metabolic adjustments in the evolution of body size, showing both shared and size‐specific patterns. BAGs are enriched in diverse metabolic processes, such as amino acid, lipid, fatty acid, and carbohydrate metabolism, supporting the idea that broad changes in core metabolic pathways accompany shifts in body mass. Large‐bodied mustelids show positive selection in pathways related to mitochondrial gene expression, fatty acid metabolism, and oxidoreductase activity, reflecting heightened energetic demands and potentially altered metabolic efficiency. Enrichment for mitochondrial translation and RNA processing genes suggests adaptations for enhanced energy production capacity. In contrast, small‐bodied mustelids exhibit positive selection in genes associated with enhanced catabolic processes, which may enable more efficient nutrient utilization in species with higher mass‐specific metabolic rates. Our identification of 149 body size‐associated genes (BAGs) highlights pathways central to growth regulation, including insulin, FoxO, and AMPK signaling, consistent with studies linking hormonal regulation and energy metabolism to body size plasticity across mammals (Bernstein 2010; Sun et al. 2019). Our findings on energy metabolism‐related genes align with the established relationship between body size and diet, as larger animals require greater energy intake and typically exploit more abundant food resources (Price and Hopkins 2015; Soler et al. 2016).

A striking convergence across BAGs, PSGs, and REGs is observed in genes involved in cytoskeletal regulation and cellular architecture. BAGs show significant enrichment in actin cytoskeleton organization and lamellipodium assembly, processes fundamental to cell shape, migration, and tissue morphogenesis. PSGs in large‐bodied species are enriched for actin cytoskeleton organization and membrane protein localization, while those in small‐bodied species exhibit enrichment in precise cytoskeletal organization. The overlap between BAGs and REGs provides particular insight into genes that not only correlate with body mass across the mustelid phylogeny but also show accelerated evolution, specifically in large‐bodied lineages. Given that both large‐bodied species in our dataset (the giant otter and the sea otter) are aquatic or semi‐aquatic, we cannot fully exclude the possibility that some of the identified genes may be related to aquatic adaptation in addition to, or rather than, body size, as suggested by the recent finding that aquatic or semi‐aquatic otters typically have large body mass in mustelids (Harano and Kutsukake 2024). The presence of metabolic regulators among the overlapping BAGs and REGs, particularly *PRKAG3*, further emphasizes the central role of metabolic adaptation in body size evolution. These genes likely contribute to the metabolic scalability necessary for supporting diverse body sizes. The *PRKAG3* gene encodes the gamma 3 subunit of AMP‐activated protein kinase (AMPK), a crucial regulator of energy metabolism in skeletal muscle (Ryan et al. 2012). Variations in this gene have been shown to be associated with meat quality development in various livestock species, including pigs (Lindahl et al. 2004), sheep (Yang et al. 2015), cattle (Ciani et al. 2007), and chickens (Zhao et al. 2006). This is further supported by the enrichment of BAGs in insulin, AMPK, and growth hormone signaling pathways, which collectively regulate energy balance and nutrient utilization. Radixin (*RDX*), identified among both BAGs and REGs and showing strong positive selection in large‐bodied lineages, represents a particularly compelling candidate. Radixin is a cytoskeletal protein involved in linking actin to the plasma membrane, and previous studies have shown that it plays a crucial role in promoting cell migration by regulating Rac1‐mediated epithelial polarity and the formation of adherens junctions through Vav GEFs (Valderrama et al. 2012).

## 5 Conclusion

In this study, we present the first comprehensive genomic analysis of body‐size evolution in Mustelidae and show that phenotypic diversification is driven not by a handful of master regulators but by a distributed genetic architecture. Coordinated modifications in growth‐factor signaling, cytoskeletal organization, metabolic pathways, and sensory systems underlie the repeated, independent shifts in body mass across the mustelid phylogeny. By identifying candidate genes, especially those under positive selection and accelerated evolution in size‐specific lineages, we lay the groundwork for targeted functional experiments. Moreover, the concordance of our results with patterns reported in other mammalian clades suggests that similar molecular strategies may govern body‐size evolution across vertebrates, offering new insights into the fundamental principles that shape adaptive phenotypic diversity.

## Author Contributions

**Tian Xia:** data curation (equal), methodology (equal), software (equal), writing – original draft (equal), writing – review and editing (equal). **Guolei Sun:** software (equal). **Guangshuai Liu:** data curation (equal), supervision (equal). **Chao Zhao:** software (equal). **Lei Zhang:** methodology (equal). **Xibao Wang:** software (equal). **Xiufeng Yang:** supervision (equal). **Xiaoyang Wu:** software (equal). **Xiaodong Gao:** writing – original draft (equal). **Honghai Zhang:** project administration (lead), writing – review and editing (equal).

## Conflicts of Interest

The authors declare no conflicts of interest.

## Supporting information


**Table S1:** The gene list of body size–associated genes (BAGs), positively selected genes (PSGs), and rapidly evolving genes (REGs).

**Table S2:** Results of the GO functional enrichment analysis of BAGs.

**Table S3:** GO enrichment results for PSGs and REGs in large‐bodied mustelids.

**Table S4:** GO enrichment results of PSGs in small‐bodied mustelids.

## Data Availability

All genomic data used in this study is publicly available from the National Center for Biotechnology Information (NCBI) database. The specific assembly accession numbers for each genome analyzed are provided in Table 1.
